# The value of diffusion kurtosis imaging, diffusion weighted imaging and ^18^F-FDG PET for differentiating benign and malignant solitary pulmonary lesions and predicting pathological grading

**DOI:** 10.3389/fonc.2022.873669

**Published:** 2022-07-29

**Authors:** Ziqiang Li, Yu Luo, Han Jiang, Nan Meng, Zhun Huang, Pengyang Feng, Ting Fang, Fangfang Fu, Xiaochen Li, Yan Bai, Wei Wei, Yang Yang, Jianmin Yuan, Jianjian Cheng, Meiyun Wang

**Affiliations:** ^1^ Department of the Graduate Student, Xinxiang Medical University, Xinxiang, China; ^2^ Department of Medical Imaging, Henan Provincial People’s Hospital, Zhengzhou, China; ^3^ Department of Medical Imaging, Zhengzhou University People’s Hospital & Henan Provincial People’s Hospital, Zhengzhou, China; ^4^ Department of Medical Imaging, Henan Provincial People’s Hospital, Henan University People’s Hospital, Zhengzhou, China; ^5^ Beijing United Imaging Research Institute of Intelligent Imaging, Beijing, China; ^6^ Central Research Institute, United Imaging Healthcare, Shanghai, China; ^7^ Department of Respiratory and Critical Care Medicine, Henan Provincial People’s Hospital, Zhengzhou University People’s Hospital, Henan University People’s Hospital, Zhengzhou, China

**Keywords:** solitary pulmonary lesion, PET/MRI, diffusion kurtosis imaging, lung cancer, histopathological grade

## Abstract

**Objective:**

To explore the value of PET/MRI, including diffusion kurtosis imaging (DKI), diffusion weighted imaging (DWI) and positron emission tomography (PET), for distinguishing between benign and malignant solitary pulmonary lesions (SPLs) and predicting the histopathological grading of malignant SPLs.

**Material and methods:**

Chest PET, DKI and DWI scans of 73 patients with SPL were performed by PET/MRI. The apparent diffusion coefficient (ADC), mean diffusivity (MD), mean kurtosis (MK), maximum standard uptake value (SUV_max_), metabolic total volume (MTV) and total lesion glycolysis (TLG) were calculated. Student’s t test or the Mann–Whitney U test was used to analyze the differences in parameters between groups. Receiver operating characteristic (ROC) curves were used to evaluate the diagnostic efficacy. Logistic regression analysis was used to evaluate independent predictors.

**Results:**

The MK and SUV_max_ were significantly higher, and the MD and ADC were significantly lower in the malignant group (0.59 ± 0.13, 10.25 ± 4.20, 2.27 ± 0.51[×10^-3^ mm^2^/s] and 1.35 ± 0.33 [×10^-3^ mm^2^/s]) compared to the benign group (0.47 ± 0.08, 5.49 ± 4.05, 2.85 ± 0.60 [×10^-3^ mm^2^/s] and 1.67 ± 0.33 [×10^-3^ mm^2^/s]). The MD and ADC were significantly lower, and the MTV and TLG were significantly higher in the high-grade malignant SPLs group (2.11 ± 0.51 [×10^-3^ mm^2^/s], 1.35 ± 0.33 [×10^-3^ mm^2^/s], 35.87 ± 42.24 and 119.58 ± 163.65) than in the non-high-grade malignant SPLs group (2.46 ± 0.46 [×10^-3^ mm^2^/s], 1.67 ± 0.33[×10^-3^ mm^2^/s], 20.17 ± 32.34 and 114.20 ± 178.68). In the identification of benign and malignant SPLs, the SUV_max_ and MK were independent predictors, the AUCs of the combination of SUV_max_ and MK, SUV_max_, MK, MD, and ADC were 0.875, 0.787, 0.848, 0.769, and 0.822, respectively. In the identification of high-grade and non-high-grade malignant SPLs, the AUCs of MD, ADC, MTV, and TLG were 0.729, 0.680, 0.693, and 0.711, respectively.

**Conclusion:**

DWI, DKI, and PET in PET/MRI are all effective methods to distinguish benign from malignant SPLs, and are also helpful in evaluating the pathological grading of malignant SPLs.

## Introduction

Lung cancer is one of the most common types of cancer in humans and one of the most common causes of cancer-related death (1). Lung cancer most commonly manifests as a solitary pulmonary lesion (SPL) (2). There are substantial differences in treatment and prognosis between benign and malignant SPLs. In addition, the pathological grade of lung cancer is an important factor that affects prognosis (3, 4). Histopathological examination is the gold standard for the diagnosis of SPLs. However, biopsy, bronchoscopy and surgical resection are associated with complications, and surgery and general anesthesia may be contraindicated in some patients.

PET computed tomography (PET/CT) is a common noninvasive method for evaluating SPLs that provides both metabolic and morphological information (5). Maximum Standardized Uptake Value (SUV_max_) refers to the highest metabolic value in the focus; it can reflect the lesion metabolic activity. Previously SUV_max_ and related metabolic parameters showed good performance in identifying benign and malignant lesions of the palatine tonsil and benign and malignant lesions of the lung (6–10). This may be since cancer cells usually tend to supply energy *via* glycolytic metabolism compared to normal cells (Warburg effect) (11), and cancer cells also usually proliferate faster and therefore take up more glucose aggressively. SUV_max_ has a high diagnostic sensitivity in distinguishing benign from malignant SPLs. However, its specificity is not ideal. This is because some active inflammatory lesions also require large amounts of glucose for energy supply, which leads to a significant increase in FDG uptake (12). In addition, a small proportion of lung cancer has no significant increase in glucose uptake due to low proliferative activity and other reasons, which may lead to false negatives (13, 14). In addition, some studies found that SUV_max_ also helps to identify the pathological grade of lung cancer and predict the prognosis of non-small cell lung cancer (15, 16). This may be because tumors with higher pathological grade and poorer prognosis are more malignant, and cell proliferation is usually more rapid and therefore their glucose uptake is higher. Metabolic tumor volume (MTV) and total lesion glycolysis (TLG) provide metabolic and volume information about the lesion, which can reflect the overall metabolism of the lesion. However, MTV and TLG have not been evaluated in SPLs; thus, it is meaningful to explore the value of these two parameters in the evaluation of SPLs.

Unlike PET/CT, PET/MRI does not have radiation from CT, and it can provide multiple functional parameters along with morphological and metabolic information about the lesion (17–20). Our previous PET/MRI studies showed that PET imaging, intravoxel incoherent motion (IVIM) and amide proton transfer weighted imaging (APTw) are all effective methods to distinguish benign SPL from malignant SPL (21), and some parameters combined with DWI and PET may predict the lung adenocarcinoma Ki-67 proliferation index (PI) (22). In addition, one study showed that SUV_max_ in PET/MRI is a predictor of overall survival in patients with non-small cell lung cancer (23).

Diffusion weighted imaging (DWI) can reflect the microstructure of tissues by obtaining information about the diffusion of water molecules. Apparent diffusion coefficient (ADC) has good performance in the detection and characterization of pulmonary nodules or masses (24, 25). It has been reported that DWI may have a higher specificity than PET for distinguishing benign and malignant SPLs (26). In addition, ADC also has good performance for predicting the pathological grade of lung cancer (27). However, DWI is based on the Gaussian distribution model of water molecules; thus, ADC may not accurately reflect the true diffusion coefficient in human tissues (28). Diffusion kurtosis imaging (DKI) is an imaging technique based on a non-Gaussian diffusion model that detects water molecule diffusion information. DKI may reflect the tissue microstructure more accurately than DWI (29, 30). It has been reported that DKI can distinguish between benign and malignant solitary pulmonary nodules or SPLs (31, 32). In addition, DKI showed good diagnostic efficacy in differentiating the pathological grade of glioma and cervical cancer (33, 34); thus, the diagnostic effectiveness of DKI for distinguishing the pathological grade of lung cancer is worth exploring.

To the best of our knowledge, the diagnosis of SPLs by DKI combined with FDG-PET has not yet been studied. The main purpose of this study was to explore the value of PET/MRI, including DKI, DWI and PET, in distinguishing between benign and malignant SPLs and the histopathological grading of malignant SPLs.

## Materials and methods

### Study population

**Figure 1:**
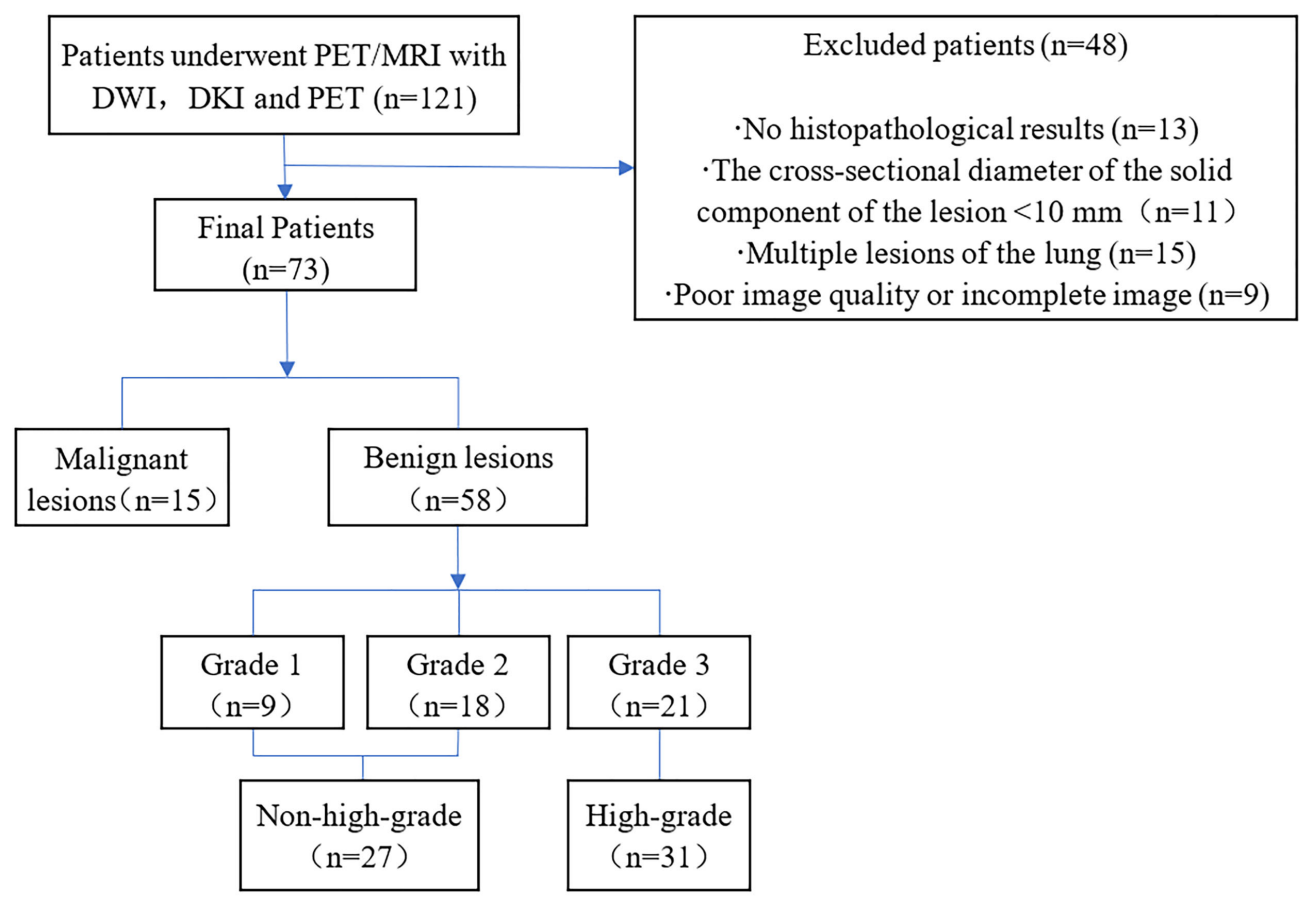
Flow diagram of the patient selection process.

From July 2020 to June 2021, a total of 121 patients with pulmonary lumps or nodules diagnosed by chest CT underwent chest PET/MRI. The inclusion criteria were as follows: 1. The lesions were not treated with invasive treatment, radiotherapy or chemotherapy before PET/MRI examination and 2. No FDG-PET/MRI contraindications (such as poor control of fasting blood glucose, ferromagnetic implants such as pacemakers, claustrophobia) were present. The following patients were excluded: 1. No histopathological results were obtained (n=13), 2. The cross-sectional diameter of the solid component of the lesion <10 mm (n = 11), 3. Multiple lesions of the lung (n=15) were present and, 4. Poor image quality or incomplete image sequences were present (n = 9). Ultimately, a total of 73 patients (53 males and 20 females; aged 60.00 ± 10.41 years; maximum diameter of lesion 3.68 ± 1.77 cm) were included in this study (**Figure 1**).

### Imaging protocol

Chest scans were performed on all patients using an integrated 3.0T PET/MRI (uPMR790, UIH, Shanghai, China) and 12-channel phased-array body coil. The tracer was ^18^F-FDG, and the injection dose was 0.11 mCi/kg. Fasting blood glucose levels were < 8.0 mmol/L after at least 6 hours of fasting before the examination. PET/MRI began 40–60 min after intravenous injection of 4.07 MBq/kg dose of ^18^F-FDG. The scan ranged from the tip of the lung to the diaphragmatic angle. In the process of PET scanning, the DixonMRI sequence was used to attenuate the gamma rays, and the ordered subset maximum expectation iteration method (OSME) was used to reconstruct the image. PET scanning (27 min) was performed in the following sequence: MR-based attenuation correction (MRAC), axial T_2_WI, axial T_1_WI, DWI, and DKI (**Table 1**).

**Table 1:** Imaging protocol parameters.

Parameters	MRAC	T2WI	T1WI	DWI	DKI
TR*(ms)	4.92	3315	4.24	1620	1210
TE* (ms)	2.24	87.8	1.13	69.8	86
Slice*thickness*(mm)	2	5	6	5	5
FOV*(mm)	500×350	500×350	500×350	500×350	500×350
Matrix	192×192	320×70	320×70	128×100	128×100
NEX	2	2	1	2, 6	1,4,8,8
b-values	/	/	/	0, 1000 s/mm^2^	0,500,1000,2000 s/mm^2^
Orientation	Axial	Axial	Axial	Axial	Axial
Breath control	Free	Navigation	Holding	Free	Free
Scanning time	2min4s	2min26s	14s	40s	6min31s

MRAC, MR-base attenuation correction; T2WI, T2-weighted imaging; T1WI, T1-weighted imaging; DWI, diffusion weighted imaging; DKI, diffusion kurtosis imaging; TR/TE, repetition time/echo time; FOV, field of view; NEX, number of excitations.

### Image processing

All PET, DKI and DWI images were imported into a United Imaging Healthcare (UIH) workstation (uWS-MR: R005) for postprocessing and measurement. The measured values of PET parameters were automatically calculated by the software, which automatically covers the whole lesion, and then the SUV_max_, MTV and TLG values were calculated. SUV_max_ is the highest SUV value of the lesion as a whole, and MTV is the volume calculated by adding all the voxels larger than the threshold value by setting the threshold of 40% SUV_max_. TLG is determined by the multiplication of MTV with average SUV. The parameters of DWI and DKI were measured by two radiologists (ZQ L and N M, with 5 and 7 years of experience in imaging diagnosis, respectively), blinded to the clinical and pathological information as well as each other’s outcomes, who analyzed all image data separately. When delineating the ROIs, the radiologists were asked to avoid areas of blood vessels, the trachea, necrosis and bleeding to ensure selection of the solid area with uniform texture.

The parameter values were calculated using the following formula, where b represents the diffusion sensitivity, S (b) is the signal strength at different b values, and S_0_ is the signal strength of b=0 s/mm². The ADC was calculated using two b values (0,1000 s/mm^2^) and a single exponential fitting model.

DWI single index ADC calculation formula:


$$\frac{S\left(b\right)}{S_{0}}=exp\left(-bADC\right)$$

where ADC is the apparent diffusion coefficient (35).

For the DKI model, the mean diffusivity (MD), mean kurtosis (MK) values are calculated by fitting the following nonlinear equations using four b values (0, 500, 1000, 2000 s/mm^2^):


$$\frac{S\left(b\right)}{S_{0}}=exp\left(-bD_{app}+\frac{1}{6}b^{2}D_{app}^{2}K_{app}\right)$$

where D_app_ and K_app_ reflect the diffusion coefficient corrected for non-Gaussian bias and the degree of deviation from the Gaussian distribution, respectively. MD and MK are the mean D_app_ and K_app_ values of all directions (36).

The lesion diameter was measured by a radiologist (Han Jiang, with 6 years of experience) on the maximum cross-section of the lesions on T_2_WI.

### Histopathological grading

According to the histological characteristics of the tumor, lung cancer was divided into three grades: Grade 1 (highly differentiated), Grade 2 (moderately differentiated) and Grade 3 (poorly differentiated). In this study, small cell lung cancer (SCLC) is a highly malignant neuroendocrine tumor; therefore, it is classified as Grade 3; bronchial adenoma is a low-grade malignant tumor, which is classified as Grade 1 in this study. Grade 3 tumors were classified as the high-grade group because of their high malignant degree, while Grade 1 and Grade 2 were classified as the non-high-grade group (27).

### Statistical analysis

All data were statistically analyzed using MedCalc 20.0 software (MedCalc Software, Mariakerke, Belgium) and SPSS 19.0 software (SPSS, Chicago, IL). The intragroup correlation coefficient (ICC) was used to evaluate the interobserver reliability of the two radiologists (ICC ≥ 0.75, excellent; 0.60 ≥ ICC < 0.75, good; 0.40 ≤ ICC < 0.60, fair; ICC < 0.40, poor) (37). Independent sample t tests or U tests were used to analyze the differences in parameters between groups. The diagnostic performance was evaluated by the ROC curve, we used the area under the curve (AUC) and the Youden index to evaluate the ROC curves, and the parameter value corresponding to the maximum value of the Youden index was set as the diagnostic cutoff value. The Delong test was used to analyze the difference in the AUCs. Logistic regression analysis was used to evaluate independent predictors and multiparametric joint diagnosis. Age, sex, smoking status, lesion diameter, and PET/MRI parameters were included in the univariate logistic regression analyses, and parameters with P < 0.1 in the analysis would be included in the multivariate logistic analysis. These tests were bilateral, and p < 0.05 was considered to be statistically significant.

## Result

### Consistency analysis

The parameters obtained by the two surveyors were in excellent agreement. The ICC values of ADC, MD and MK were 0.836, 0.897 and 0.867, respectively. In all subsequent analyses, the average values of the parameters of the two readers were used. The values of SUV_max_, MTV and TLG were calculated automatically by workstation software, and there was no need for a consistency check.

### Parameter comparison

**Figure 2:**
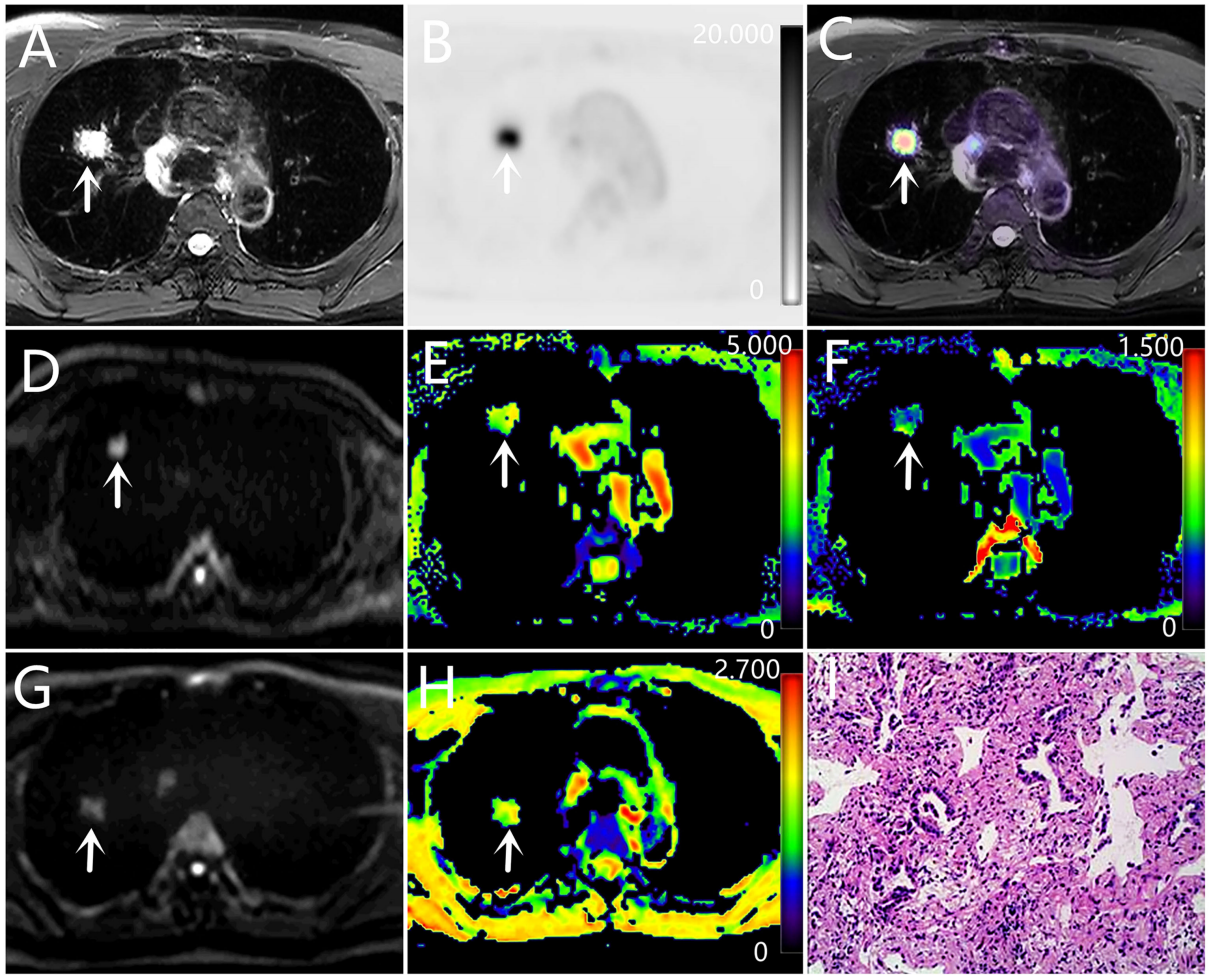
A-H: a 63-year-old male with adenocarcinoma (poorly differentiated). **(A)** T_2_WI, **(B)** PET original image, SUV_max_ = 11.94, MTV = 1.69, TLG = 12.47, **(C)** PET and T_2_WI fusion map, **(D)** DKI original image (b=1000 s/mm^2^), **(E)** MD pseudo-color map, MD = 2.54×10^-3^ mm²/s, **(F)** MK pseudo-color map, MK = 0.44 **(G)** DWI original image (b=1000 s/mm^2^), **(H)** ADC pseudo-color map, ADC = 1.43×10^-3^ mm²/s, **(I)** hematoxylin and eosin (H&E) staining image.

**Figure 3:**
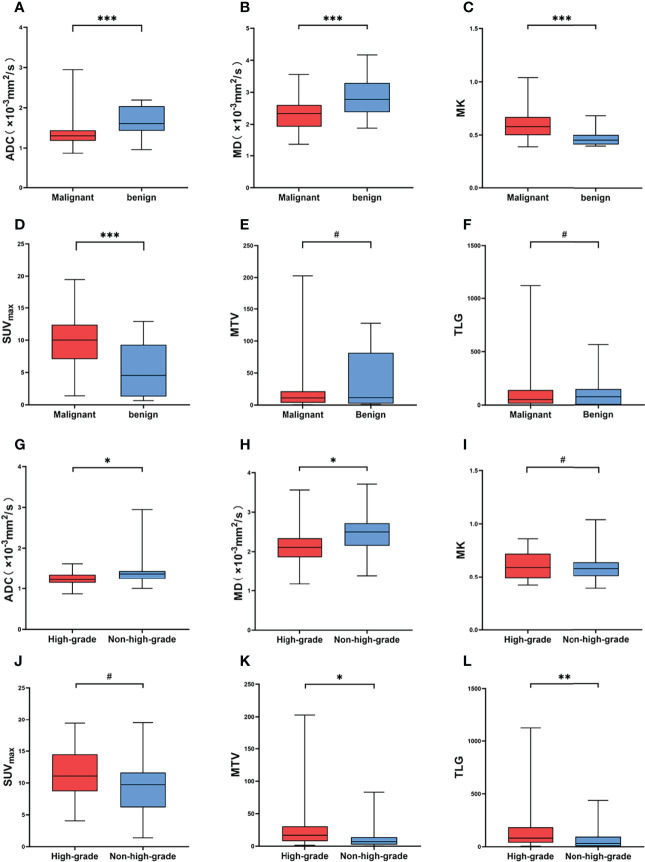
**(A–F)** Comparison of different parameters between malignant and benign SPL groups. **(G–L)** Comparison of different parameters between high-grade and non-high-grade in malignant SPL group. # Represents P > 0.05, * represents P < 0.05, ** represents P < 0.01, *** represents P < 0.001.

The MK and SUV_max_ values of malignant SPLs were significantly higher than those of benign SPLs, while the values of MD and ADC were significantly lower than those of benign SPLs. There was no significant difference in MTV or TLG between the two groups (P = 0.594 and 0.548, respectively). The values of ADC and MD in high-grade malignant SPLs were significantly lower than those in non-high-grade SPLs, while the values of MTV and TLG were significantly higher than those in non-high-grade SPLs. There were no significant differences in MK and SUV_max_ values between the high-grade group and the non-high-grade group (P = 0.653 and 0.083, respectively) (**Table 2** and **Figures 2**, **3**).

**Table 2:** Comparison of different parameters among different groups.

Parameters	Malignant	Benign	p value	High-grade	Non-high-grade	p value
ADC(*10^-3^mm²/s)	1.35 ± 0.33	1.67 ± 0.33	**<0.001^b^**	1.35 ± 0.33	1.67 ± 0.33	**0.019^b^**
MD(*10^-3^mm²/s)	2.27 ± 0.51	2.85 ± 0.60	**<0.001^a^**	2.11 ± 0.51	2.46 ± 0.46	**0.010^a^**
MK	0.59 ± 0.13	0.47 ± 0.08	**<0.001^b^**	0.60 ± 0.13	0.59 ± 0.12	0.653^a^
SUV_max_	10.25 ± 4.20	5.49 ± 4.05	**<0.001^a^**	11.14 ± 4.20	9.22 ± 4.08	0.083^a^
MTV	20.17 ± 32.34	35.87 ± 42.24	0.594^b^	35.87 ± 42.24	20.17 ± 32.34	**0.012^b^**
TLG	114.20 ± 178.68	119.58 ± 163.65	0.548^b^	119.58 ± 163.65	114.20 ± 178.68	**0.006^b^**

The bold in the table represents a statistically significant comparison. ^a^means independent t test is used for comparison, and ^b^means Mann-Whitney U test is used for comparison.

### Regression analysis

Smoking, sex, age, lesion diameter and related parameters were included in the analysis. The univariate logistic regression analysis showed that age, ADC, MD, MK and SUV_max_ were predictive factors for differentiating benign from malignant SPLs, but in the multivariate analysis, only MK and SUV_max_ were independent predictors (**Table 3**).

**Table 3:** Univariate and multivariate analysis of differentiating benign and malignant solitary pulmonary lesions.

Parameters	Univariate Analyses		Multivariate Analyses	
	OR (95%CI)	P-value	OR (95%CI)	P-value
Smoking	1.091 (0.342-3.484)	0.883	/	/
Gender	0.342 (0.070-1.675)	0.186	/	/
Age (year)	1.060 (1.003-1.120)	0.040	1.322 (0.636-2.748)	0.454
lesion diameter (cm)	0.942 (0.687-1.294)	0.713	/	/
ADC (×10^-3^mm^2^/s)	0.099 (0.017-0.564)	0.009	0.603 (0.268-1.357)	0.222
MD (×10^-3^mm^2^/s)	0.149 (0.044-0.500)	0.002	1.025 (0.386-2.722)	0.961
MK	7.191 (2.044-25.297)	0.002	4.523 (1.029-19.877)	**0.046**
SUV_max_	1.353 (1.130-1.622)	0.001	2.974 (1.121-7.891)	**0.029**
MTV	0.989 (0.975-1.004)	0.144	/	/
TLG	1.000 (0.997-1.003)	0.915	/	/

All factors with P < 0.1 in univariate analyses were included in multivariate regression analyses. The bold typeface in the table indicates the logistic regression analyses with statistical significance. OR, odds ratio; CI, confidence interval.

### Diagnostic performance of different parameters

**Figure 4:**
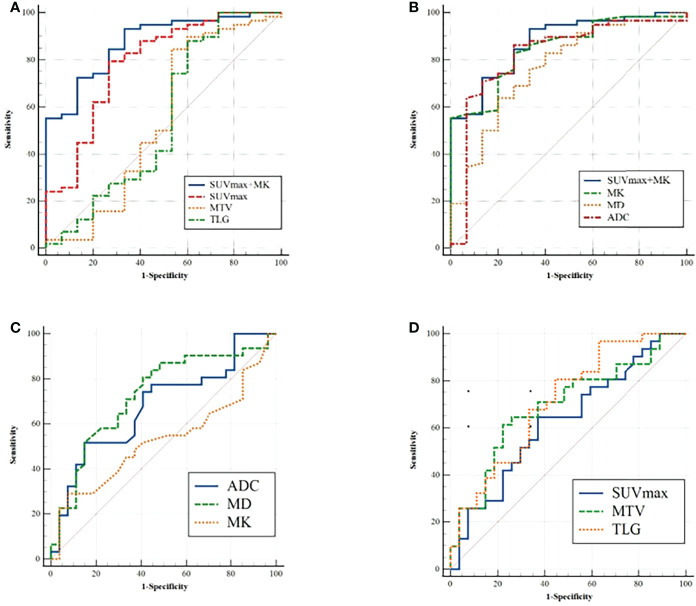
**(A, B)** ROC analysis for differentiation of malignant and benign groups; **(C, D)** ROC analysis for differentiation of high-grade and non-high-grade groups.

In distinguishing benign from malignant SPLs, the independent predictors SUV_max_ and MK were used for combined diagnostics. The AUCs of SUV_max_+MK, SUV_max_, MK, MD and ADC were 0.875, 0.787, 0.848, 0.769 and 0.822, respectively. AUC (MK+SUV_max_) > AUC (MK) > AUC (ADC) > AUC (SUV_max_) > AUC (MD). However, only the difference between AUC (MK+SUV_max_) and AUC (MD) was statistically significant (P = 0.0444). In terms of distinguishing between the high-level and low-level groups of malignant SPLs, the AUCs of MD, ADC, MTV and TLG were 0.729, 0.680, 0.693 and 0.711, respectively, but there were no significant differences (**Tables 4**, **5** and **Figure 4**).

**Table 4:** Sensitivity and specificity of diffusion parameters at optimal cutoff values in differentiating malignant from benign solitary pulmonary lesions.

Parameters	AUC (95% CI)	P-Value	Youden Index		Cutoff value	Sensitivity (%)	Specificity (%)	Comparison With Combined Diagnosis
SUV_max_+MK	0.875(0.776-0.941)	<0.0001	0.5977	–		93.10	66.67	/
SUV_max_	0.787(0.676-0.874)	0.0001	0.5264	6.33		79.31	73.33	P=0.1244
MK	0.826(0.720-0.905)	<0.0001	0.5437	0.47		81.03	73.33	P=0.5608
MD(×10^-3^mm^2^)	0.769(0.656-0.860)	0.0001	0.4276	3.31		53.03	86.67	P=0.0444
ADC(×10^-3^mm^2^)	0.822(0.715-0.902)	<0.0001	0.5954	1.5		86.21	73.33	P=0.5045
MTV	0.545(0.424-0.662)	0.6692	0.3115	/		/	/	/
TLG	0.551(0.430-0.667)	0.6240	0.2793	/		/	/	/

The combined diagnosis represents SUV_max_ + MK. The differences of AUC between the combination of SUV_max_ + MK, and MD were significant (P < 0.05). The differences of AUC among SUV_max_+MK, MK, ADC and SUV_max_ were not statistically significant.

**Table 5:** Sensitivity and specificity of diffusion parameters at optimal cutoff values in differentiating high-grade and non-high-grade malignant SPLs.

Parameters	AUC (95% CI)	P-Value	Youden Index	Cutoff value	Sensitivity (%)	Specificity (%)
MK	0.529 (0.393-0.661)	0.7125	0.2162	/	/	/
MD (×10^-3^mm^2^)	0.729 (0.596-0.837)	0.0008	0.3990	2.35	80.65	59.26
ADC (×10^-3^mm^2^)	0.680 (0.545-0.797)	0.0116	0.3680	0.47	81.03	73.33
SUV_max_	0.628 (0.491-0.751)	0.0857	0.2748	/	/	/
MTV	0.693 (0.558-0.807)	0.0063	0.3907	13.76	61.29	77.78
TLG	0.711 (0.577-0.822)	0.00221	0.3620	33.95	80.65	55.56

AUC, Area under the curve; MK, mean kurtosis; SUV_max_, maximum standard uptake value; MD, mean diffusivity.

## Discussion

SUV_max_ reflects the highest metabolic activity of pixels/voxels in the lesion. In the present study, the SUV_max_ of malignant SPLs was higher than that of benign SPLs, which is consistent with previous data (13), indicating that the proliferative activity of malignant SPL cells is higher than that of benign SPLs. MTV and TLG combine information on the metabolic burden and volume of the lesion. Nakajo et al. showed that MTV and TLG can identify benign and malignant lung lesions (38). In contrast, in our study, there was no significant difference in MTV or TLG between the benign and malignant groups of SPLs. The inconsistent results of the studies may be due to different inclusion criteria and different PET imaging devices; Nakajo et al. included multiple lung lesions in their study and the imaging device they used was PET/CT. These two results need to be confirmed by further large sample studies. In our study, there was no significant difference in SUV_max_ between high-grade and non-high-grade malignant SPLs, which was contrary to the results of previous studies evaluating the differentiation of lung adenocarcinoma by SUV_max_ (15, 39). One reason for the inconsistent results may be that the previous research object was identifying the pathological type of lung adenocarcinoma, and tumor cell proliferation modes were similar, while this study included a variety of pathological types, and the tumor cell proliferation modes differed. In addition, the MTV and TLG in the high-grade group were significantly higher than those in the non-high-grade group. This may be due to the higher aggressiveness of tumor cells in high-grade lung cancer, leading to increased glucose uptake by the tumor, resulting in higher MTV and TLG values. The prognosis of high-grade lung cancer may be poor due to its high aggressiveness. The previous finding has shown that high MTV and TLG values were independently associated with shorter overall survival in lung cancer patients (40), and our finding was consistent with their result.

Both ADC and MD can reflect the limited diffusion of water molecules in tissue, which is mainly affected by cell density (27). In this study, the ADC and MD values of malignant SPLs were lower than those of benign SPLs, which was consistent with the results of previous studies (31, 32, 41). However, the results of Uto et al. (42) and Koyama et al. (25) showed no significant difference in ADC values between benign and malignant pulmonary nodules or lesions. This inconsistency may be due to the different b values used and the different lesion inclusion and exclusion criteria. It has been reported that b values lower than 600 s/mm^2^ are affected by perfusion effects (42). In addition, studies have shown that the detection rate of DWI for < 5 mm pulmonary nodules is less than 50%, while for > 10 mm pulmonary nodules, it is as high as 97% (43). In this study, the b value selected by DWI was (0, 1000 s/mm^2^), and the inclusion standard of lesions was SPLs with the cross-sectional diameter of the solid component of the lesion > 10 mm, which probably reduced the interference of other factors to some extent. In the present study, the high-grade ADC and MD values of malignant SPLs were significantly lower than those of non-high-grade SPLs, which was consistent with previous studies (27, 44). The reason may be that the higher the tumor grade is, the denser the tissue structure will be, resulting in the increased limitation of water molecule diffusion and lower ADC and MD values.

MK is mainly related to the complexity of tissue structure in many diseases (29). In this study, the MK value of the malignant SPL group was significantly higher than that of the benign group, which was consistent with the results of Das et al. (32). This may be due to the higher heterogeneity and irregularity of malignant SPL tissue, the larger number of interfaces in the tissue, and the increased limitation of water diffusion. In addition, MK was an independent predictor of benign and malignant SPLs in this study; however, the results of Wan et al. showed that there was no significant difference in MK between benign and malignant SPLs (31), and the choice of b value may be one of the reasons for the inconsistent results. Wan et al. also included pulmonary inflammatory lesions with an MK value as high as 2.37, which may have affected the results. In this study, there was no significant difference in the MK value between high-grade and non-high-grade malignant SPLs, which indicates that the tissue complexity of high-grade lung cancer is not necessarily high, but this finding may also be due to the inclusion of a variety of pathological types of lung cancer in this study.

In terms of the diagnostic efficiency of distinguishing benign from malignant SPLs, the AUC of SUV_max_+MK was the largest, but it was only significantly different from the AUC of MD, and there was no significant difference with the AUC of SUV_max_ or MK, which may indicate that the combination (SUV_max_+MK) does not significantly improve the diagnostic efficiency of differential SPLs. There was no significant difference in AUCs among single parameters (ADC, MD, MTV and TLG) for distinguishing high-grade and non-high-grade lung cancer, which indicated that DWI, DKI and PET were all helpful for the pathological grading of malignant SPLs, but none of those parameters showed higher diagnostic efficiency.

This study has some limitations. First, there were relatively few cases of benign SPLs, squamous cell carcinoma and small cell carcinoma in this study, and there was also a lack of other malignant histological types (such as single metastasis and lung carcinoid). This is due to the nature of prospective studies, where subjects undergoing PET/MRI are usually suspected of having lung malignancy and have a higher incidence of lung adenocarcinoma than other lung cancer subtypes. Second, both DWI and DKI are echo-planar imaging (EPI)-based sequences, which make it difficult to evaluate minimal lesions due to the low signal-to-noise ratio, low spatial resolution, and susceptibility to artifacts of EPI. Third, the ROIs of DWI and DKI avoid necrosis, cystic degeneration or vascular areas, which may not be conducive to a comprehensive evaluation of tumor tissue structure. This is due to the large variation in these areas between lesions, which can interfere significantly with the parameter values. Fourth, Since the main purpose of this study was to evaluate the diagnostic efficacy of PET/MRI, there was no comparison with PET/CT, which may make this study less informative for clinical applications. Finally, this study is a limited cohort single-center analysis, which may have resulted in selection bias. In future studies, we will continue to expand the sample size and try to reduce the impact of these limitations through several methods.

## Conclusion

DWI, DKI, and ^18^F-FDG PET imaging in PET/MRI are all effective methods to distinguish benign from malignant SPLs and are also helpful in the diagnosis of pathological grading of malignant SPLs. The combination of the independent predictors SUV_max_ and MK had higher diagnostic efficacy than MD in differentiating benign from malignant SPLs.

## Data availability statement

The original contribution presented in this study are included in the article/supplementary material. Further inquiries can be directed to the corresponding author.

## Ethics statement

The studies involving human participants were reviewed and approved by ethics committee of Henan Provincial People’s Hospital. The patients/participants provided their written informed consent to participate in this study.

## Author contributions

MW: Conceptualization, Funding acquisition, Methodology, Writing-Reviewing and Editing, Validation. JC: Conceptualization, Methodology, Writing-Reviewing and Editing. ZL: Data curation, Methodology, Formal analysis, Writing-Original draft preparation. NM: Methodology, Writing- Reviewing and Editing. YL: Formal analysis, Investigation. HJ: Formal analysis, Writing-Original draft preparation. ZH: Methodology, Investigation. TF: Investigation. PF: Investigation. FF: Project administration, Investigation, Funding acquisition. YB: Project administration, Investigation. WW: Project administration, Investigation. YY: Software, Writing-Reviewing and Editing. JY: Software. All authors contributed to the article and approved the submitted version.

## Funding

This work was supported by the National Key R&D Program of China [grant number 2017YFE0103600], the National Natural Science Foundation of China [grant number 81720108021 and 31470047]; and the Zhengzhou Collaborative Innovation Major Project [grant number 20XTZX05015]; and the Henan provincial science and technology research projects [grant number 212102310689], and Key Project of Henan Province Medical Science and Technology Project (LHGJ20210001).

## Conflict of interest

JY and YY are employees of United Imaging Healthcare (UIH).

The remaining authors declare no relationships with any companies whose products or services may be related to the subject matter of the article.

## Publisher’s note

All claims expressed in this article are solely those of the authors and do not necessarily represent those of their affiliated organizations, or those of the publisher, the editors and the reviewers. Any product that may be evaluated in this article, or claim that may be made by its manufacturer, is not guaranteed or endorsed by the publisher.
